# Primary Epstein-Barr Virus Infection Presenting as Cholestatic Hepatitis Without Infectious Mononucleosis in an Immunocompetent Middle-Aged Adult: A Case Report

**DOI:** 10.7759/cureus.96907

**Published:** 2025-11-15

**Authors:** Mirac Burak Tak, Annabelle Li Kam Wa, Lauren Simkin, Mohamed Abdelnabi, Anika Tasneem

**Affiliations:** 1 Internal Medicine, University Hospitals Sussex NHS Foundation Trust, Worthing, GBR

**Keywords:** atypical presentation, cholestatic liver injury, ebv, ebv-associated hepatitis, ebv hepatitis, epstein-barr virus, infectious mononucleosis, jaundice cholestatic, viral hepatitis, viral hepatitis differential

## Abstract

Epstein-Barr virus (EBV) infections commonly present with a subclinical hepatitis, often in the context of infectious mononucleosis (IM). A 56-year-old male presented with fever, alongside signs of jaundice and right upper quadrant tenderness. Initial investigations revealed marked cholestatic hepatic dysfunction, but imaging showed no evidence of biliary tree pathology. Serological and molecular testing confirmed primary EBV infection, whilst excluding other viral and autoimmune causes of hepatitis. The patient was managed conservatively, with rapid normalisation of liver function during outpatient follow-up. The rare occurrence of cholestatic hepatitis secondary to primary EBV infection in an immunocompetent middle-aged adult, and the lack of typical IM symptoms - including rash, pharyngitis, and lymphadenopathy - makes diagnosis challenging and complex. Given the rare but potentially fatal risk of progression to acute liver failure, and the atypical presentation, EBV should be high on the list of differentials in cases of acute cholestatic hepatitis in the absence of other IM features, particularly in middle-aged and older adults. Once common aetiologies have been excluded, early targeted EBV serological and molecular tests are key to avoiding unnecessary invasive investigations and inappropriate management.

## Introduction

Epstein-Barr virus (EBV) is a ubiquitous human γ-herpesvirus that infects more than 90% of the worldwide population, typically establishing lifelong latency after primary infection [1]. EBV seroprevalence increases with age from 1-4.9 years (68%-72%) to 20-25 years (95%-96%) in the United Kingdom [2]. Most primary EBV infections occur in childhood and follow an asymptomatic course, whereas infections in adolescents and young adults classically present as infectious mononucleosis (IM), with fever, pharyngitis, and lymphadenopathy. Less common manifestations include EBV-associated autoimmune and lymphoproliferative disorders [1,3]. Liver involvement in EBV infection is common, typically presenting with transient, mildly elevated aminotransferases, with cholestatic hepatitis reported in only 5% of cases [4-6]. It is even rarer to observe acute, symptomatic EBV hepatitis without classical IM features [7]. EBV hepatitis can mimic autoimmune or other viral hepatitis and can be easily overlooked if not included in the list of differentials. This case describes an unusual presentation of isolated, EBV-induced cholestatic hepatitis in an immunocompetent middle-aged adult. It highlights the diagnostic complexities encountered and the importance of targeted viral panels in investigating unexplained cholestatic liver injury.

This case was previously presented in abstract form as a poster at the Royal College of Physicians Medicine 2025 conference, and the abstract was published in Clinical Medicine (Journal of the Royal College of Physicians) in July 2025.

## Case presentation

A 56-year-old male presented to the Emergency Medicine Department with a 10-day history of right upper quadrant pain, fever, rigours, malaise, nausea, anorexia, and dark urine. He denied any sore throat, rash, mass, or weight loss. 

The patient’s past medical history was significant for gout, hypercholesterolemia, and hypertension. There was no personal or family history of liver disease, viral hepatitis, or autoimmune conditions. Regular medications included atorvastatin 20 mg OD and allopurinol 300 mg OD; the latter, which he had discontinued three months prior to presentation. He denied the use of any immunosuppressants, corticosteroids, biologics, over-the-counter or herbal medications, recent antibiotics, or hepatotoxic medications. His childhood vaccinations were up to date. He consumed one to two alcoholic drinks socially on weekends, with no history of excessive alcohol intake. He was sexually active with a regular partner and denied any high-risk sexual activity or sexually transmitted infections. He denied a history of recent travel, unwell contacts, smoking, recreational drug use, tattoos or piercings, blood transfusions, or occupational exposure to toxins. 

On examination, the patient was jaundiced with tenderness in the right upper quadrant, but there was no cervical lymphadenopathy, tonsillitis, oral mucosal lesions, nor cutaneous rash.

Blood tests revealed raised liver enzymes, bilirubin, and ferritin (Table 1). Lymphocyte counts had increased on Day 3, remained raised until Day 23, and then gradually normalised. C-reactive protein (CRP) levels were only mildly to moderately raised.

**Table 1:** Summary of biochemical investigations Abbreviations: ALT - alanine aminotransferase; AST - aspartate aminotransferase; ALP - alkaline phosphatase; GGT - gamma-glutamyl transferase; LDH - lactate dehydrogenase; INR - international normalised ratio; TIBC - total iron-binding capacity; UIBC - unsaturated iron-binding capacity; IFCC - International Federation of Clinical Chemistry and Laboratory Medicine; DCCT - Diabetes Control and Complications Trial; WBC - white blood cells; MCV - mean corpuscular volume; MCH - mean corpuscular haemoglobin; MCHC - mean corpuscular haemoglobin concentration; eGFR - estimated glomerular filtration rate; CRP - C-reactive protein; HDL - high-density lipoprotein; LDL - low-density lipoprotein

Test	Units	Reference Range	Baseline (Two months ago)	Day 1	Day 2	Day 3	Day 4	Day 5	Day 6	Day 7	Day 8	Day 9	Day 10	Day 11	Day 12	Day 22	Day 26	Day 33	Day 37	Day 40	Day 43	Day 64	Day 71
Liver Function Tests																							
ALT	U/L	10-45	27	264	237	236	190	205	265	-	340	320	258	232	197	183	212	219	165	91	57	47	43
AST	U/L	10-40	-	238	-	204	-	-	-	-	-	-	324	284	-	191	-	-	-	54	36	-	-
ALP	U/L	30-130	68	373	388	387	336	297	300	-	390	426	439	442	416	430	326	278	188	126	98	92	81
GGT	U/L	10-60	-	-	-	-	-	-	-	-	-	429	-	-	561	-	-	116	83	73	65	33	-
LDH	U/L	140-280	-	-	-	-	-	-	-	-	-	455	-	-	-	-	-	-	-	-	-	-	-
Conjugated Bilirubin	µmol/L	1.0-4.0	-	-	-	-	-	-	-	-	-	160	-	-	-	-	-	-	-	-	-	-	-
Unconjugated Bilirubin	µmol/L	3.0-13	-	-	-	-	-	-	-	-	-	31	-	-	-	-	-	-	-	-	-	-	-
Conjugated/Total Bilirubin Ratio	-	-	-	-	-	-	-	-	-	-	-	0.84	-	-	-	-	-	-	-	-	-	-	-
Total Bilirubin	µmol/L	3-21	9	63	152	153	160	168	-	181	203	191	204	188	197	160	62	52	38	29	27	25	11
Albumin	g/L	35-50	46	40	38	38	33	29	29	-	25	25	22	22	20	21	31	34	36	41	39	41	43
Total Protein	g/L	60-80	76	78	74	74	70	64	66	-	65	69	66	66	63	67	89	93	91	95	89	92	90
Globulin	g/L	20-35	30	38	36	36	37	35	37	-	40	44	44	44	43	46	58	59	55	54	50	51	47
INR	-	0.8-1.2	-	-	1.1	-	1.1	-	-	-	1.1	1.1	1	1	1	1	0.9	-	0.9	0.9	1	1	1
Iron Studies																							
Ferritin	µg/L	30-400	-	2344	-	-	-	-	-	-	-	-	-	-	-	-	-	-	367	-	-	-	-
Transferrin Saturation	%	20-45	-	10	22	-	-	-	-	-	-	-	-	-	-	-	-	-	-	-	-	-	-
Transferrin	g/L	2.0-3.6	-	2.3	2.1	-	-	-	-	-	-	-	-	-	-	-	-	-	-	-	-	-	-
Iron	µmol/L	10-30	-	5.9	11.6	-	-	-	-	-	-	-	-	-	-	-	-	-	-	-	-	-	-
TIBC	µmol/L	45-72	-	57.7	52.7	-	-	-	-	-	-	-	-	-	-	-	-	-	-	-	-	-	-
UIBC	µmol/L	20-62	-	51.8	41.1	-	-	-	-	-	-	-	-	-	-	-	-	-	-	-	-	-	-
HbA1c																							
HbA1c IFCC	mmol/mol	<42	39	-	-	-	-	-	-	-	-	-	-	-	-	-	-	-	34	-	-	-	-
HbA1c DCCT	% (DCCT units)	<6.0	5.7	-	-	-	-	-	-	-	-	-	-	-	-	-	-	-	5.3	-	-	-	-
Full Blood Count and CRP																							
Haemoglobin	g/L	130-170	140	161	148	140	125	129	-	121	131	124	129	118	124	143	137	141	132	139	137	138	-
Lymphocytes	×10⁹/L	1.0-4.0	2.2	1.6	1.3	2.2	3.8	5.3	-	5.2	3.9	3.8	4.8	3.8	4.8	4.2	3.7	2.5	2	1.7	2	1.9	-
Neutrophiles	×10⁹/L	1.5-8.0	4	3.1	3.3	3.3	3.3	4	-	3.3	3.5	2.4	3.1	2.3	2.5	1.1	1.3	1.4	2.2	3.4	2.2	3.3	-
CRP	mg/L	<5	1	31	43	-	42	-	-	-	20	-	11	-	6	-	-	-	-	-	-	-	-
WBC	×10⁹/L	4.0-11.0	7.1	5.4	5.2	6.6	8.2	10.6	-	9.7	8.4	7.2	9.2	7.7	8.2	6.4	5.9	4,8	5.1	5,9	5	6.1	-
Platelets	×10⁹/L	150-400	244	152	140	132	143	118	-	129	155	157	155	141	173	244	153	118	213	255	166	228	-
Haematocrit	Fraction	0.40-0.50	0.42	0.47	-	0.39	0.35	0.37	-	0.36	0.38	0.36	0.38	0.35	0.37	0.44	0.42	0.42	0.39	0.4	0.41	0.41	-
MCV	fL	80-100	94	90	89	88	89	90	-	91	89	92	90	90	92	89	87	88	87	87	86	87	-
MCH	pg	27-33	30.9	31.3	31.3	31.4	31.6	31.6	-	30.9	30.6	31.3	30.5	30.5	30.4	29.1	28.8	29.2	29.3	29.8	29.1	29.4	-
MCHC	g/L	320-360	331	347	354	358	356	351	-	341	344	342	340	339	332	328	330	334	339	344	337	336	-
Renal Profile																							
Creatinine	µmol/L	60-110	73	79	-	61	-	66	64	74	-	61	57	59	54	-	68	70	66	70	69	73	-
eGFR	mL/min/1.73m²	≥60	>90	>90	-	>90	-	>90	>90	>90	-	>90	>90	>90	>90	-	>90	>90	>90	>90	>90	>90	-
Urea	mmol/L	2.5-7.8	-	6	-	4.2	-	4.1	4.3	4.6	-	3.4	3.3	2.9	2.8	-	-	-	-	-	-	-	-
Sodium	mmol/L	135-145	136	132	-	129	-	131	129	130	-	130	130	130	126	-	132	133	135	136	137	136	-
Potassium	mmol/L	3.5-5.1	4.6	4.3	-	4	-	3.8	3.8	3.7	-	4	4	3.6	3.7	-	4.8	4.6	4.6	4.5	4.5	4.2	-
Chloride	mmol/L	98-107	-	99	-	98	-	95	95	95	-	96	94	98	96	-	-	-	-	-	-	-	-
Lipid Profile																							
Cholesterol	mmol/L	2.0-5.0	-	-	2.61	-	-	-	-	-	-	-	-	-	-	-	-	-	-	-	-	-	-
Triglyceride	mmol/L	0.5-2.5	-	-	2.5	-	-	-	-	-	-	-	-	-	-	-	-	-	-	-	-	-	-
HDL	mmol/L	>1.0	-	-	0.34	-	-	-	-	-	-	-	-	-	-	-	-	-	-	-	-	-	-
LDL	mmol/L	0-2.5	-	-	1.33	-	-	-	-	-	-	-	-	-	-	-	-	-	-	-	-	-	-
Non-HDL	mmol/L	<4.0	-	-	2.27	-	-	-	-	-	-	-	-	-	-	-	-	-	-	-	-	-	-

Autoimmune screen showed a positive smooth muscle antibody (SMA) on Day 2 and a subsequent negative result on Day 9 (Table 2). Anti-Ro52 blot was weakly positive on Day 9, while other autoimmune serology, including antinuclear antibody (ANA), liver-kidney microsomal antibody (LKM), soluble liver antigen (SLA), anti-mitochondrial antibody (AMA), anti-glycoprotein 210 (anti-gp210), anti-promyelocytic leukaemia protein (anti-PML), anti-speckled protein 100 (anti-Sp100), and connective tissue disease screen, were negative. The titres for positive autoimmune panels were unavailable to the authors. Immunoglobulin testing demonstrated a polyclonal pattern with elevated immunoglobulin A (IgA), immunoglobulin M (IgM), immunoglobulin G (IgG), and immunoglobulin E (IgE) levels. An elevated Fibrosis-4 (FIB-4) score was noted.

**Table 2:** Summary of autoimmune, immunoglobulin, protein, and fibrosis assessment results Abbreviations: Ag - antigen; Ab - antibody; SLA - soluble liver antigen; LKM - liver kidney microsomal; Ro52 - anti-Ro52 antibody; gp210 - glycoprotein 210; PML - promyelocytic leukaemia protein; Sp100 - speckled protein 100; IgG - immunoglobulin G; IgA - immunoglobulin A; IgM - immunoglobulin M; IgE - immunoglobulin E

Test	Units	Reference Range	Baseline	Day 1	Day 2	Day 3	Day 4	Day 5	Day 6	Day 7	Day 8	Day 9
Proteins												
A1-antitrypsin	g/L	0.9-2.0	-	-	2.05	-	-	-	-	-	-	-
Caeruloplasmin	g/L	0.2-0.6	-	-	0.47	-	-	-	-	-	-	-
Alpha-feto protein	µg/L	<10	-	-	<1.7	-	-	-	-	-	-	-
Autoimmune Screen												
Anti-nuclear antibody	Qualitative	Negative	-	-	Negative	-	-	-	-	-	-	Negative
Anti-SLA blot	Qualitative	Negative	-	-	-	-	-	-	-	-	-	Negative
Anti-LKM blot	Qualitative	Negative	-	-	Negative	-	-	-	-	-	-	Negative
Anti-Ro52 blot	Qualitative	Negative	-	-	-	-	-	-	-	-	-	Weak Positive
Anti-gp210 ab	Qualitative	Negative	-	-	-	-	-	-	-	-	-	Negative
Anti-PML ab	Qualitative	Negative	-	-	-	-	-	-	-	-	-	Negative
Anti-Sp100 ab	Qualitative	Negative	-	-	-	-	-	-	-	-	-	Negative
Anti-M2-3E ab	Qualitative	Negative	-	-	-	-	-	-	-	-	-	Negative
Connective tissue disease screen	Qualitative	Negative	-	-	-	-	-	-	Negative	-	-	Negative
Smooth muscle ab	Qualitative	Negative	-	-	Positive	-	-	-	-	-	-	Negative
Anti-mitochondrial ab	Qualitative	Negative	-	-	Negative	-	-	-	-	-	-	Negative
Immunoglobulins												
IgG	g/L	7-16	-	12.6	13.6	-	-	-	-	-	19.9	18.3
IgA	g/L	0.7-4.0	-	4.1	4.31	-	-	-	-	-	6.63	6.49
IgM	g/L	0.4-2.3	-	1.71	2.03	-	-	-	-	-	2.95	2.76
IgE	IU/mL	<100 IU/mL	-	-	-	-	-	-	-	-	-	529
Fibrosis Assessment												
Fibrosis-4 Score	N/A	<1.3: low risk; >2.67: high risk	5.35	-	-	-	-	-	-	-	-	-

Serological and molecular testing for EBV was performed in a United Kingdom Accreditation Service-accredited National Health Service virology laboratory compliant with international standards. The specific assay platforms and cut-off values used to define seropositivity were not available to the authors. EBV serology results were reported qualitatively for EBV viral capsid antigen (VCA) IgM, VCA IgG, and EBV nuclear antigen (EBNA) IgG. EBV DNA levels were reported quantitatively as the number of viral copies per millilitre of blood. Initial EBV-specific serological and molecular test results on Day 2 were suggestive of primary EBV infection: EBV VCA IgM was positive, VCA IgG was negative, and EBNA IgG was negative (Table 3). EBV DNA was 141,705 copies/mL. On Day 9, VCA IgG became positive, while VCA IgM remained positive, and EBNA IgG remained negative. EBV DNA decreased to 31,000 copies/mL.

Other viral causes of acute hepatitis, including hepatitis A-E, were excluded (Table 3). Cytomegalovirus (CMV) IgG was positive, whilst CMV IgM and CMV DNA were negative, consistent with previous exposure. Adenovirus DNA was negative. Herpes simplex virus (HSV) testing was not performed. 

**Table 3:** Summary of microbiological investigations Abbreviations: WBC - white blood cells; RBC - red blood cells; MRSA - methicillin-resistant *Staphylococcus aureus*; Hep - hepatitis; Ag - antigen; Ab - antibody; IgM - immunoglobulin M; IgG - immunoglobulin G; HIV - human immunodeficiency virus; CMV - cytomegalovirus; EBV - Epstein-Barr virus; VCA - viral capsid antigen; DNA - deoxyribonucleic acid

Test	Reference Range	Day 1	Day 2	Day 3	Day 4	Day 5	Day 9	Day 12
Urine								
WBC	Not detected	Trace	-	-	-	-	-	-
RBC	Not detected	Not detected	-	-	-	-	-	-
Epithelial cells	Not detected	Trace	-	-	-	-	-	-
Culture	No growth	No growth	-	-	-	-	-	-
MRSA								
Nose swab	Negative	-	-	Negative	Negative	-	-	-
Blood Culture								
Aerobic/anaerobic growth	No growth	-	-	No growth	-	No growth	No growth	-
Hepatitis								
Hep A IgM	Negative	Negative	-	-	-	-	-	-
Hep B Surface Ag	Negative	Negative	-	-	-	-	-	-
Hep C Ab	Negative	Negative	-	-	-	-	-	-
Hep E IgG/IgM	Negative	-	-	-	-	Negative/Negative	-	-
HIV								
HIV Ag/Ab	Not detected	-	Not detected	-	-	-	Not detected	-
CMV								
IgG	Negative	-	Positive	-	-	-	Detected	-
IgM	Negative	-	Negative	-	-	-	Not detected	-
DNA	Not detected	-	-	-	-	Not detected	-	Not detected
EBV								
VCA IgM	Negative	-	Positive	-	-	-	Positive	-
VCA IgG	Negative	-	Negative	-	-	-	Positive	-
Nuclear Ag IgG	Negative	-	Negative	-	-	-	Negative	-
DNA (copies/mL)	Not detected	-	141,705	-	-	-	35,420	15,003
Adenovirus								
DNA	Not detected	-	-	-	-	Not detected	-	Not detected

**Figure 1:**
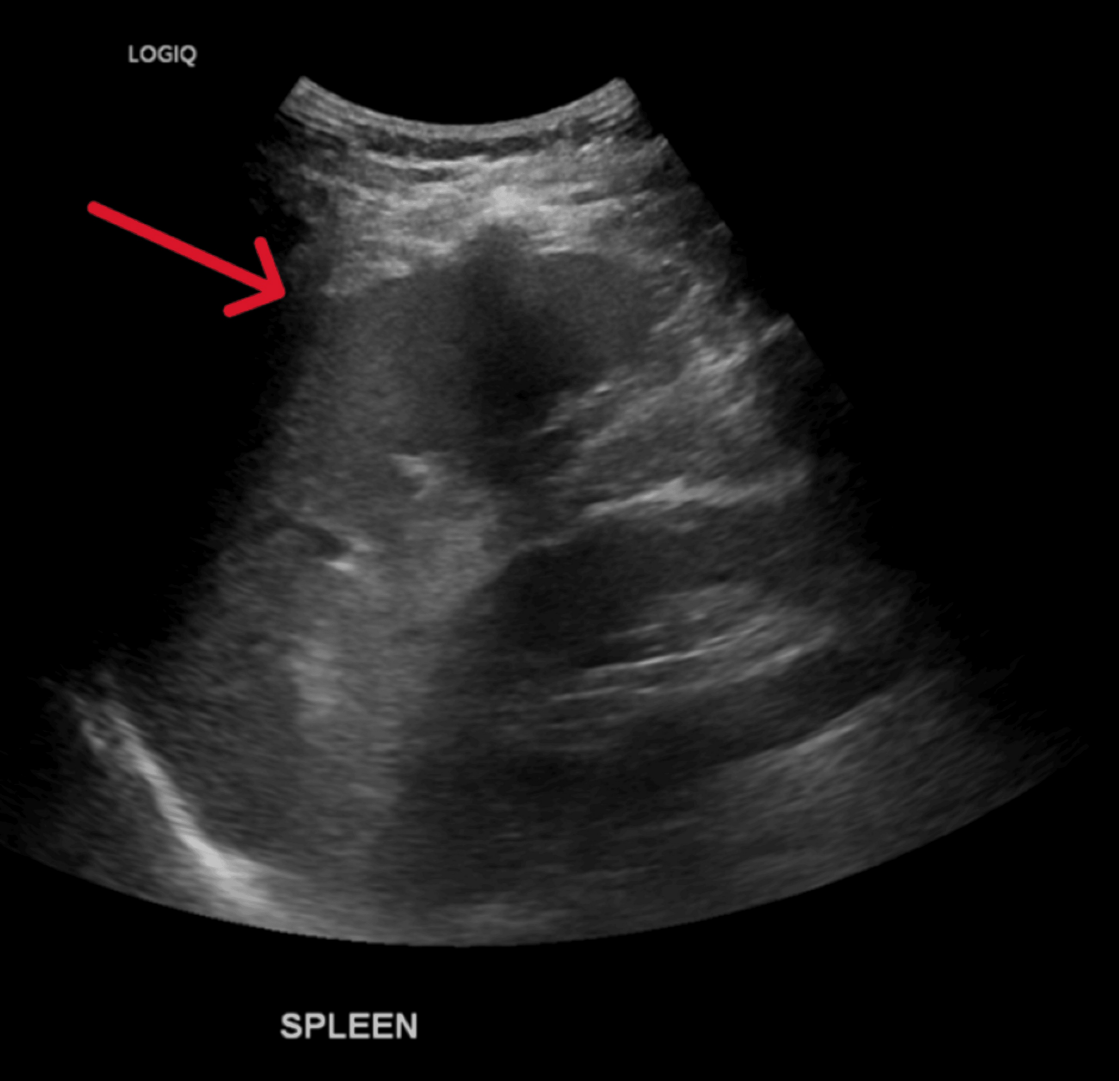
Ultrasound of the abdomen showing mild splenomegaly (red arrow)

**Figure 2:**
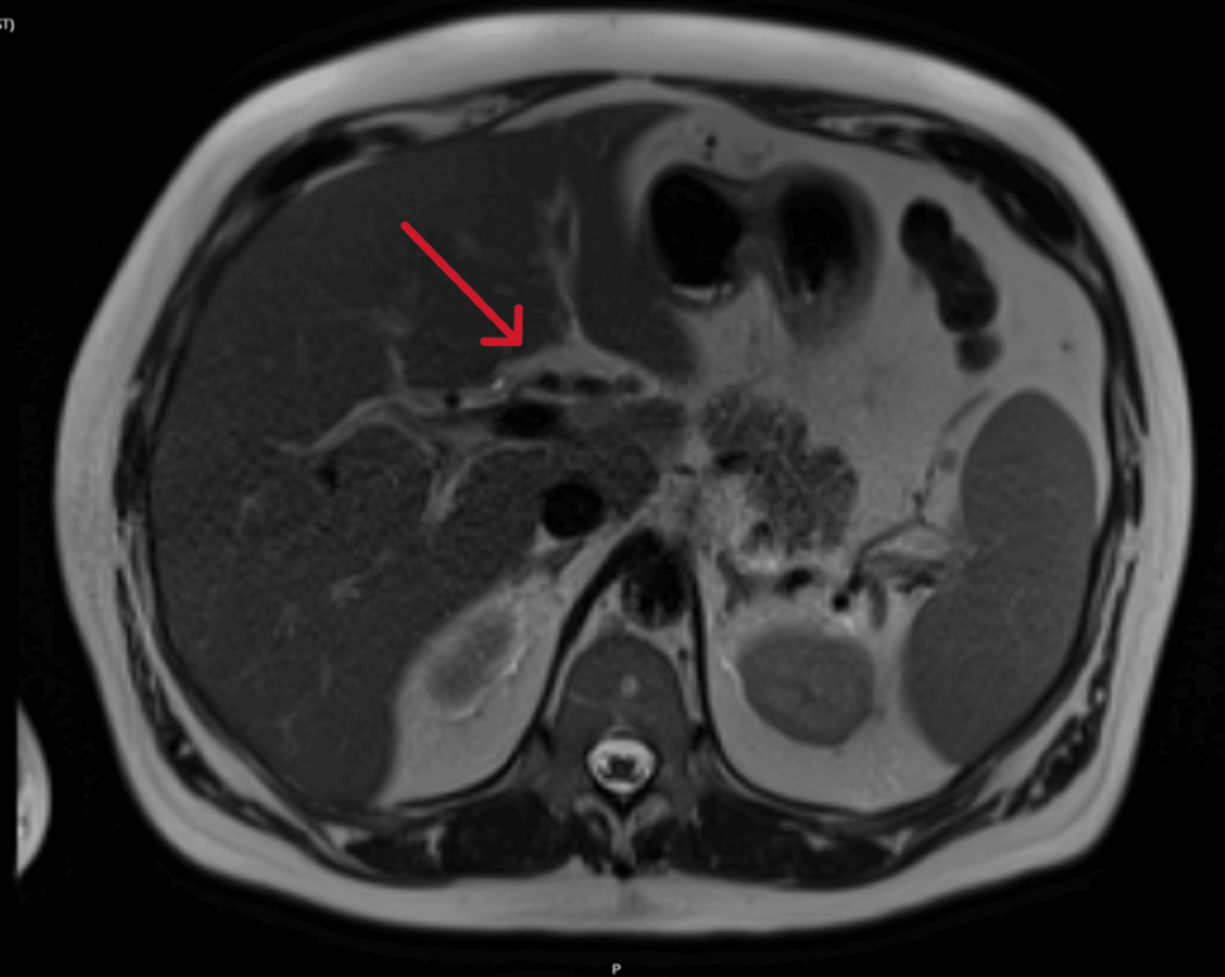
MRCP demonstrating subtle periportal oedema (red arrow) Abbreviation: MRCP - magnetic resonance cholangiopancreatography

**Figure 3:**
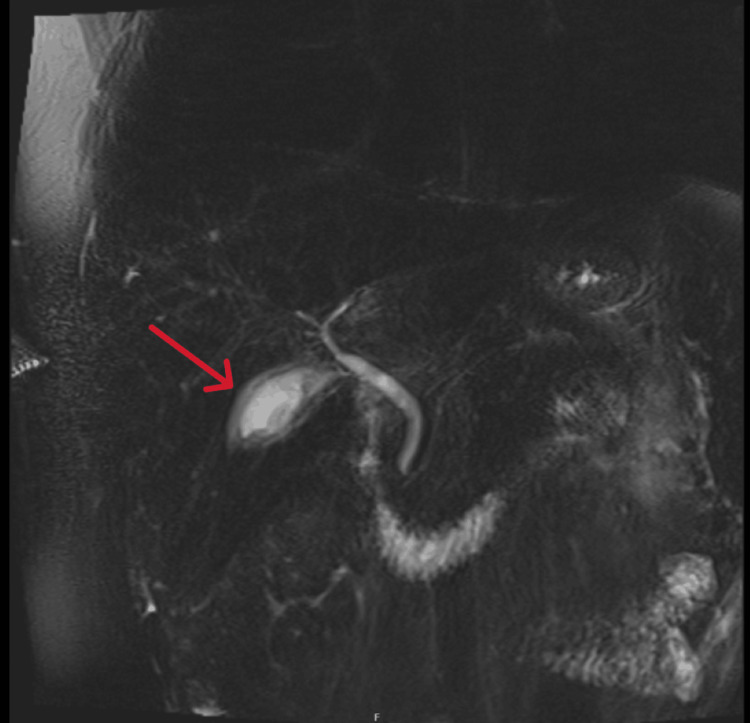
MRCP demonstrating marked gallbladder wall thickening and oedema (red arrow) Abbreviation: MRCP - magnetic resonance cholangiopancreatography

Ultrasound revealed mild splenomegaly (Figure 1). Magnetic resonance cholangiopancreatography (MRCP) demonstrated marked gallbladder wall thickening with oedema and subtle periportal oedema. The liver contour was unremarkable. There were no pericholecystic inflammatory changes, gallstones, or biliary dilatation. These findings, in the absence of pericholecystic inflammation or biliary obstruction, were interpreted by the radiology team as a reactive finding secondary to acute hepatitis, rather than cholecystitis or obstructive pathology (Figures 2-3).

Liver biopsy was considered to exclude autoimmune or alternative causes, but was not pursued, as the risks outweighed the benefits, given the rapid improvement in symptoms and liver enzymes. 

**Figure 4:**
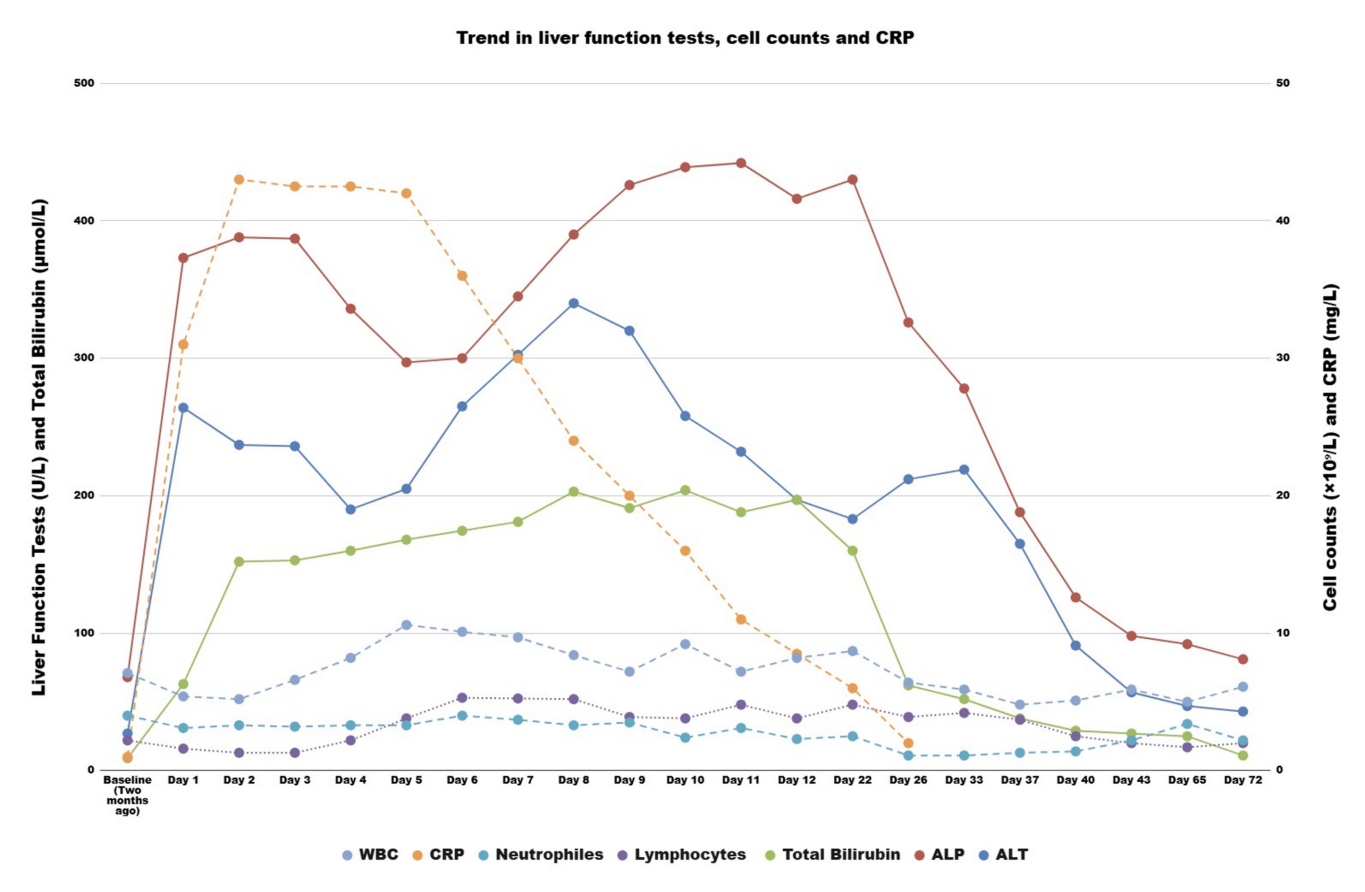
Trend in liver function tests, cell counts, and CRP Continuous lines should be interpreted with the reference ranges indicated on the left y-axis, whereas dashed lines should be interpreted with the reference ranges indicated on the right y-axis. Abbreviations: ALT - alanine aminotransferase; ALP - alkaline phosphatase; WBC - white blood cells; CRP - C-reactive protein

The patient was managed supportively with intravenous fluids and cyclizine for nausea, with close monitoring for acute liver failure (ALF) over a 12-day inpatient admission. He became afebrile after Day 3, and his abdominal pain, nausea, and anorexia gradually resolved during admission. Follow-up assessments demonstrated normalisation of liver function tests (LFTs), total bilirubin, cell counts, and CRP (Figure 4).

## Discussion

EBV-IM-related acute cholestatic hepatitis is an uncommon but known phenomenon [8-11]. Isolated EBV hepatitis without the typical features of IM is rare, with a handful of similar cases in the medical literature [7,12,13], and has been observed to occur more frequently in middle-aged and older adults ​[14,15]​.

This case illustrates an atypical presentation of primary EBV infection in an immunocompetent middle-aged adult, consistent with existing reports that deviate from the classical presentation of IM in younger adults with pharyngitis and lymphadenopathy, and that are often associated with mild, self-limiting transaminitis [14-16]. The presentation was even more unusual due to the presence of clinically evident jaundice, as, although hyperbilirubinaemia occurs in nearly half of EBV hepatitis cases, clinically evident jaundice is reported in fewer than 5% [16].

The presence of lymphocytosis, mild transient neutropenia, mild transient thrombocytopenia, jaundice, and splenomegaly supported the diagnosis of EBV infection [16,17]. Additionally, imaging findings of gallbladder wall thickening and periportal oedema, in the absence of pericholecystic inflammation or biliary obstruction, were consistent with EBV-associated hepatitis [16].

The diagnosis of primary EBV infection was confirmed by positive EBV VCA IgM, negative EBV VCA IgG, negative EBNA, and a high quantitative EBV DNA load, with EBV VCA IgG seroconversion within a week. EBV VCA IgM antibodies typically become detectable at or shortly after the onset of clinical symptoms, usually within the first week of illness, and decline to undetectable levels within several weeks. EBV VCA IgG antibodies appear slightly later during the acute phase and persist lifelong, indicating previous infection. EBNA IgG antibodies develop after the acute phase, and their presence indicates prior exposure rather than acute infection. Thus, the combination of positive VCA IgM with seroconversion of VCA IgG and negative EBNA strongly supports a diagnosis of primary EBV infection, rather than reactivation or past exposure. Elevated EBV DNA titres in plasma indicate active viral replication and can help differentiate acute infection from latency, where viral DNA levels are typically low or undetectable. In our case, the high EBV DNA load was suggestive of acute infection [18].

Another member of the Herpesviridae family, HSV, can also cause hepatitis. However, this is considered unlikely in our case, given the absence of immunosuppression, coagulopathy, thrombocytopenia, encephalopathy, and the patient’s rapid, spontaneous improvement without antiviral therapy, as HSV hepatitis has been observed to progress aggressively to ALF if left untreated [16,19,20]. CMV usually causes hepatitis in immunosuppressed or liver transplant patients, and serological testing for CMV - being IgG positive, IgM negative, and CMV DNA negative - suggested past infection with immunity [16,21].

Although an initial positive result for SMA raised concerns about autoimmune hepatitis, several findings argued against this condition: a subsequent negative SMA result within one week, rapid clinical improvement without immunosuppressive therapy, polyclonal hypergammaglobulinemia rather than isolated IgG elevation, negative ANA, absence of autoimmune history, and the presence of acute EBV infection - a known cause of viral hepatitis [22]. These features suggest that autoimmune hepatitis was unlikely, and that the SMA positivity represented a false positive or EBV-related epiphenomenon, as EBV can induce transient production of autoantibodies, including SMA and ANA [5], similar to the transient AMA positivity reported in another case [18]. Such serologic mimicry contrasts with true autoimmune hepatitis, which typically demonstrates persistent IgG elevation [3,5,19].

The elevated FIB-4 score (Table 2) was interpreted as reflecting acute hepatocellular injury rather than chronic fibrosis [23]. This was supported by the subsequent normalisation of LFTs and the MRCP findings, which showed no evidence of hepatic fibrosis.

The trend in ferritin, from initial marked elevation to normalisation within one month (Table 1), was consistent with a reactive inflammatory response to EBV. Although hyperferritinaemia is a feature of haemophagocytic lymphohistiocytosis (HLH), with EBV being the most common infectious trigger of secondary HLH [24], the clinical course did not demonstrate sepsis or multiorgan failure, coagulopathy, marked cytopenia in two or more cell lines, hypertriglyceridaemia, hepatomegaly, or marked splenomegaly; and, lastly, the rapid clinical and biochemical resolution without immunosuppression was not in keeping with HLH [24,25].

In our case, the LFTs normalised spontaneously within one month, consistent with reports of EBV hepatitis, in which liver function typically normalises within two to six weeks, reflecting the generally benign course of the disease in immunocompetent individuals [11,16]. EBV-related ALF can rarely occur, accounting for 0.2% of adult cases in a United States registry [26]. Therefore, clinicians should remain vigilant for any clinical or biochemical deterioration.

The mainstay of treatment for EBV hepatitis is supportive care. However, in severe cases, antiviral therapy and corticosteroids may be considered, with liver transplantation reserved for cases that do not improve despite medical therapy [16].

Early targeted serological and molecular EBV testing can prevent unnecessary invasive diagnostics, such as liver biopsy or endoscopy; inappropriate treatments, such as steroids for presumed autoimmune hepatitis; or withholding crucial medications for suspected drug-induced liver injury [5].

## Conclusions

Acute EBV infection should be included in the differentials for acute cholestatic hepatitis, even when characteristic IM features are absent, particularly in middle-aged and older adults. This case adds to the limited evidence that primary EBV infection can present in an immunocompetent middle-aged adult as an isolated cholestatic hepatitis, with clinically evident jaundice. Clinicians should perform targeted serological and molecular testing for EBV, guided by the clinical and biochemical pattern in acute unexplained cholestatic hepatitis, once common aetiologies have been excluded, to avoid unnecessary invasive investigations and inappropriate management. 
